# IL-1 Beta—A Biomarker for Ischemic Stroke Prognosis and Atherosclerotic Lesions of the Internal Carotid Artery

**DOI:** 10.3390/medicina59101790

**Published:** 2023-10-08

**Authors:** Maria-Gabriela Catană, Ioan-Adrian Popențiu, Mădălina Văleanu, Corina Roman-Filip, Romeo-Gabriel Mihăilă

**Affiliations:** 1 Faculty of Medicine, Lucian Blaga University of Sibiu, Izvorului Street, 550169 Sibiu, Romania; 2 Neurology Department, Emergency County Clinical Hospital Sibiu, Corneliu Coposu bvd, 550245 Sibiu, Romania; 3 Department of General Surgery, “Alexandru Augustin” Military Emergency Hospital, Victoriei bvd, 550024 Sibiu, Romania; 4 Department of Medical Informatics and Biostatistics, University of Medicine and Pharmacy “Iuliu Hatieganu” Cluj-Napoca, 7 Horea Street, 400174 Cluj-Napoca, Romania; 5 Hematology Department, Emergency County Clinical Hospital Sibiu, Sibiu Corneliu Coposu bvd, 550245 Sibiu, Romania

**Keywords:** inflammation, stroke, atheromatosis, biomarker, interleukin-1 beta

## Abstract

*Background and Objectives:* Stroke is a leading cause of mortality and morbidity worldwide. Treatment of this pathology is still under development and its risk factors remain to be determined. Therefore, we aim to determine the role of interleukin-1 beta in atherosclerotic lesions of the internal carotid artery as a risk factor for stroke and the role of this biomarker in stroke prognosis. *Materials and Methods*: This study enrolled 56 patients diagnosed with ischemic stroke in the anterior vascular territory (AVT) and posterior vascular territory (PVT). All the patients had venous blood collected at admission and 7 days after the onset of the cerebral ischemia in order to determine the plasma concentration of interleukin-1 beta. At the same time, an extracranial carotid ultrasound was performed. *Results*: The interleukin-1 beta collected at admission was positively correlated with the NIHSS at admission (Pearson index 0.424), and both measurements were correlated with carotid stenosis (Spearmen correlation index of 0.529 and 0.653, respectively). *Conclusions*: Interleukin-1 beta could be a reliable biomarker for stroke prognosis and the development of atherosclerotic lesions of the internal carotid.

## 1. Introduction

Ischemic stroke represents the third cause of worldwide morbidity and mortality, with more than 80% of the patients suffering an episode of cerebral ischemia remaining with a life-altering disability. For these patients, the short therapeutic window available for a complete treatment addressing cerebral ischemia has led to important research on a molecule able to limit the destruction of cerebral tissue [1]. In recent years, the role of neuroinflammation in neurologic pathology has been studied, highlighting proinflammatory cytokines such as interleukin-1 (IL-1), IL-6, and Tumor Necrosis Factor–alpha (TNF-alpha). IL-1 is a proinflammatory cytokine important in local and systemic inflammation, making it a central factor in immune reaction to infections and other injuries [2,3]. The IL-1 family has three main ligands: IL-1 alpha and IL-1 beta as agonists and the internal antagonist IL-1Ra. Recent studies have demonstrated that IL-1 is quickly expressed in the cerebral tissue during a neuronal injury, associated with multiple inflammatory changes [4,5]. On the other hand, other risk factors strongly related to cerebral ischemic episodes, such as cerebral and atheromatosis of the carotid artery and post-stroke infections, are correlated with strong expression of inflammatory markers, namely, nod-like receptor family pyrin domain containing 3 (NLRP3), IL-1 beta, TNF alpha, and IL6 inflammasome. Considering all this, the inflammation process will be the next therapeutic target in the future [6,7].

## 2. Materials and Methods

### Study Design

This study occurred over 12 months, between the 1st of January and the 31st of December 2021. The patients enrolled in the study were patients with acute neurological pathology and ischemic stroke less than 24 h old that had been admitted and treated in the Neurology Clinic of the Sibiu County Emergency Hospital. At admission, the biological markers were determined, with a re-evaluation 7 days after the stroke’s onset. During hospitalization, we performed ultrasound examination of the carotid arteries in order to determine the extent of the atheromatosis at this level.

The inclusion criteria were as follows:

- Adult patients (from 33 to 94 years old) admitted to the Neurology Department, at Sibiu County Emergency Hospital, with diagnostic suspicion of ischemic stroke.
- Neurological symptoms and signs strongly suggesting the onset of an ischemic stroke (within the last 24 h).
- Cerebral imaging ruling out cerebral tumors or hemorrhagic stroke.

The exclusion criteria were the following:

- Any medical pathology that can trigger modification of inflammatory markers, such as infections, autoimmune diseases, neoplasia, and hematological disorders (lymphoma amd multiple myeloma).
- Patients undergoing (in the last 30 days) corticosteroid or immunosuppressive therapy.
- Patients diagnosed over the last 180 days with acute myocardial infarction, myocarditis, or acute ischemic stroke.
- Patients who suffered brain traumatic injuries documented at the time of admission.

The term atherosclerosis is defined as the thickening of the intimal layers of the arteries and the accumulation of fat. The plaques are formed by a central core of fatty material covered by a fibrous cap. Plaques can have different sizes, thus obstructing even up to more than 70% of the vessel’s lumen [8,9]. Atherosclerotic lesions consist of two parts: atherosis, which refers to the accumulation of fat, and sclerosis, which comprises the fibrotic layer together with smooth muscle cells, leucocytes, and macrophages, driven by an innate and acquired immune response [10].

a. Collection of biological samples and bio-marker measurement

All the samples were collected in EDTA vacutainers, centrifuged at 1500× for 15 min, and frozen at −80 °C afterward. The IL-1 beta (pg/mL) biomarker was determined from these samples. The non-standard samples were excluded. The human interleukin-1 beta (IL-1β) was measured with an ELISA assay, which employs a quantitative sandwich enzyme immunoassay technique. Cholesterol was analyzed routinely using a gas chromatography (GC)-flame ionization detector without derivatization.

We used the Carotid Doppler ultrasound as the imaging tool, with the staging of carotid atheromatosis as follows:

0. Mild atherosclerotic lesions of the carotid artery.
1. Moderate atherosclerotic lesions of the carotid artery; internal carotid artery (ICA) stenosis 50–69%.
2. Severe atherosclerotic lesions of the carotid artery; internal carotid artery (ICA) stenosis greater than 70%.

b. Statistical analysis

The database was created using Microsoft Office Excel 2016. SPSS 25.0 (SPSS Inc., Chicago, IL, USA) was used for statistical analysis and data description. The normality of the distribution of quantitative data was verified using the Shapiro–Wilk or Kolmogorov–Smirnov tests. The accepted error threshold was α = 0.05. To describe the normally distributed continuous quantitative data, the arithmetic mean ± standard deviation was used, and for those that did not have a Gaussian distribution, the median (quartile 1-quartile 3) was used. The qualitative data were described using frequencies. To compare the means of the quantitative variables of two independent groups, Student’s test (*t*-test) was used if the variables were normally distributed. The nonparametric Mann–Whitney and Kruskall–Wallis tests were used to compare the means of two independent groups where the variables had an abnormal distribution. Correlation analysis was performed using the Pearson linear correlation coefficient for data with a normal distribution and the Spearman correlation coefficient for quantitative data without a normal distribution or for ordinal data. Colton’s empirical rules were used to interpret the correlation coefficients.

## 3. Results

**Figure 1:**
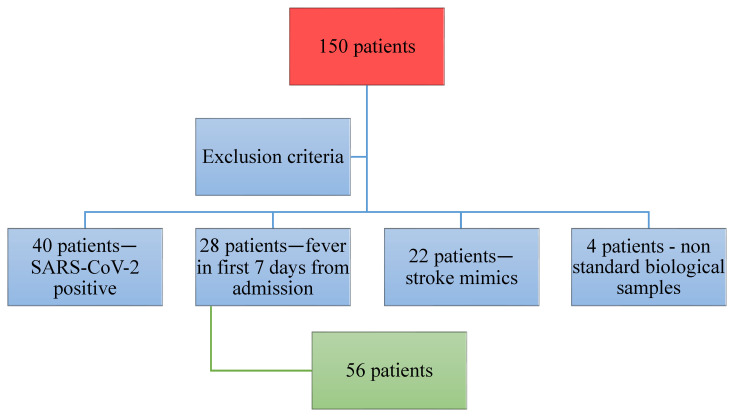
Description of the patients excluded and enrolled in the study.

The study was conducted between the 1 January 2021 and the 31 December 2021. A total of 150 patients were included in our study. Of this initial group, 40 were diagnosed with SARS-CoV-2 infection at admission, and 28 developed a fever in the first 7 days that made the second evaluation of inflammatory markers useless; for 22 patients, the diagnosis was a stroke-like syndrome, and 4 patients were excluded because of non-standard biological samples or improper harvesting and storage of sample. Overall, 56 patients remained enrolled until the end-point of the study (Figure 1).

Of the 56 patients who fulfilled the inclusion criteria, 28 were males and 28 were females, with a mean age of 74. At admission and right before discharge, the NIHSS (National Institute of Health Stroke Scale) values were determined by the neurologist. The values at admission were 24 at the highest and 2 at the lowest, while, at discharge, the values decreased for most of the patients, with a maximum of 20 and a minimum of 0. The IL-1 beta values were determined in the first 24 h after admission and on the 7th day of hospitalization.

The results of the Doppler ultrasound examination were classified into three main categories:

1. Mild atherosclerotic lesions of the carotid artery.
2. Moderate atherosclerotic lesions of the carotid artery; internal carotid artery (ICA) stenosis 50–69%.
3. Severe atherosclerotic lesions of the carotid artery; internal carotid artery (ICA) stenosis greater than 70%.

The median age of stroke patients was 74, ranging from 33 to 91 years. In addition, 80% of the patients suffered an ischemic stroke localized in the anterior vascular territory; the remaining 20% of the patients instead suffered an ischemic stroke localized in the posterior vascular territory. A total of thirty-two of the patients (76%) included in the study and diagnosed with moderate or severe atherosclerotic lesions of the internal carotid were of normal weight (BMI 18.5–24.9); two patients (5%) were overweight (BMI 25–29.9); six patients (14%) were obese (BMI > 30); and two patients (5%) were underweight (BMI < 18.5).

The median IL-1 beta plasma concentration (quartile 1–quartile 3) on day 1 was 0.51 (ranging from 0 to 1.56), and, on day 7, at the onset of the stroke, it was 0 (ranging from 0 to 1.59). The median NIHSS at admission was seven–50% of the patients had an NIHSS lower than seven, and 50% of the patients included in the study had an NIHSS greater than seven (ranging from four to eleven). A total of 25% of the patients included in the study had an NIHSS greater than eleven at admission. The median NIHSS at discharge was five (ranging from two to ten), meaning that 25% of the patients included in the study were discharged with an NIHS score greater than ten, which indicates moderate or severe disability.

An extracranial carotid ultrasound was performed on all the patients admitted to the study: a moderate to severe carotid stenosis was described in 69.6% of the subjects; 35.7% of them presented moderate stenosis (50–60% stenosis); and 33.9% of the patients had severe carotid stenosis (>70%) (Table 1).

**Figure 2:**
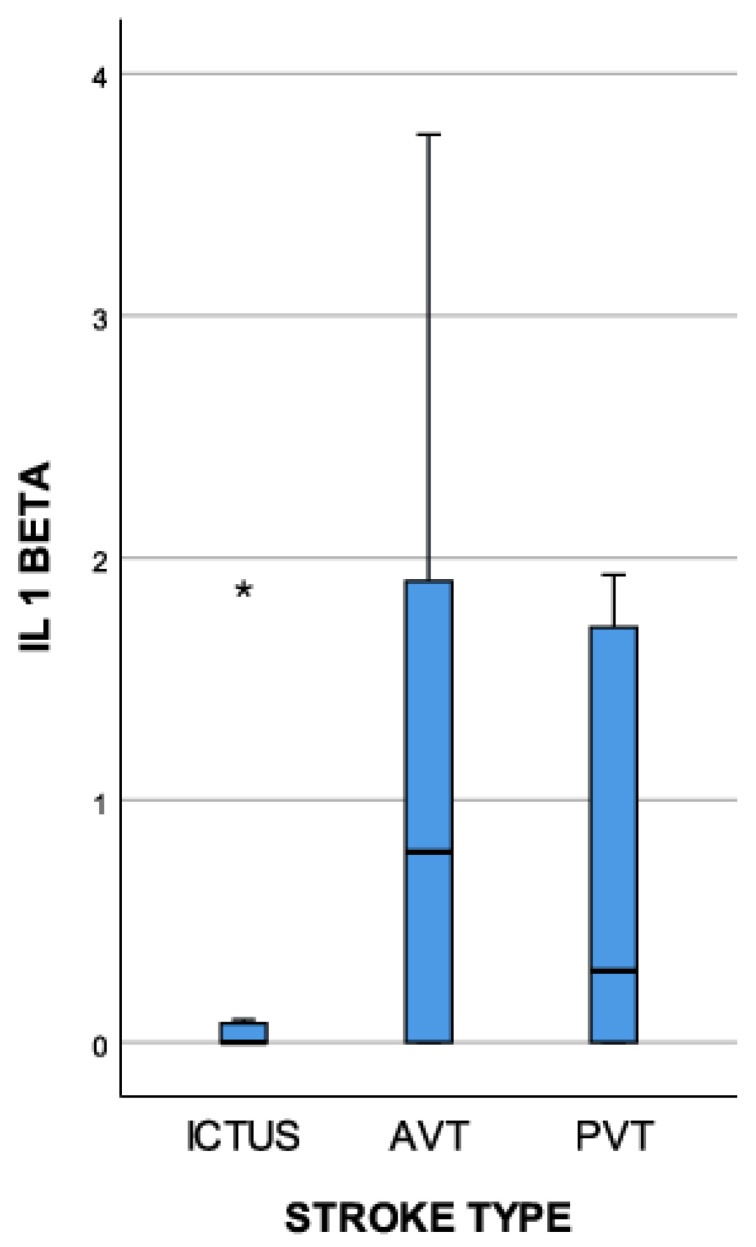
The IL-1 beta plasma concentration (pg/mL)’s correlation with the stroke type (extreme outliers are marked with an asterisk (*) on the boxplot).

As can be noted in Figure 2, the IL-1 beta value was positively correlated with the stroke type, with the plasma concentration being greater in the AVT strokes. Additionally, the IL-1 beta’s first measurement concentration was correlated with the NIHSS score at admission, with a Pearson index of 0.424.

**Figure 3:**
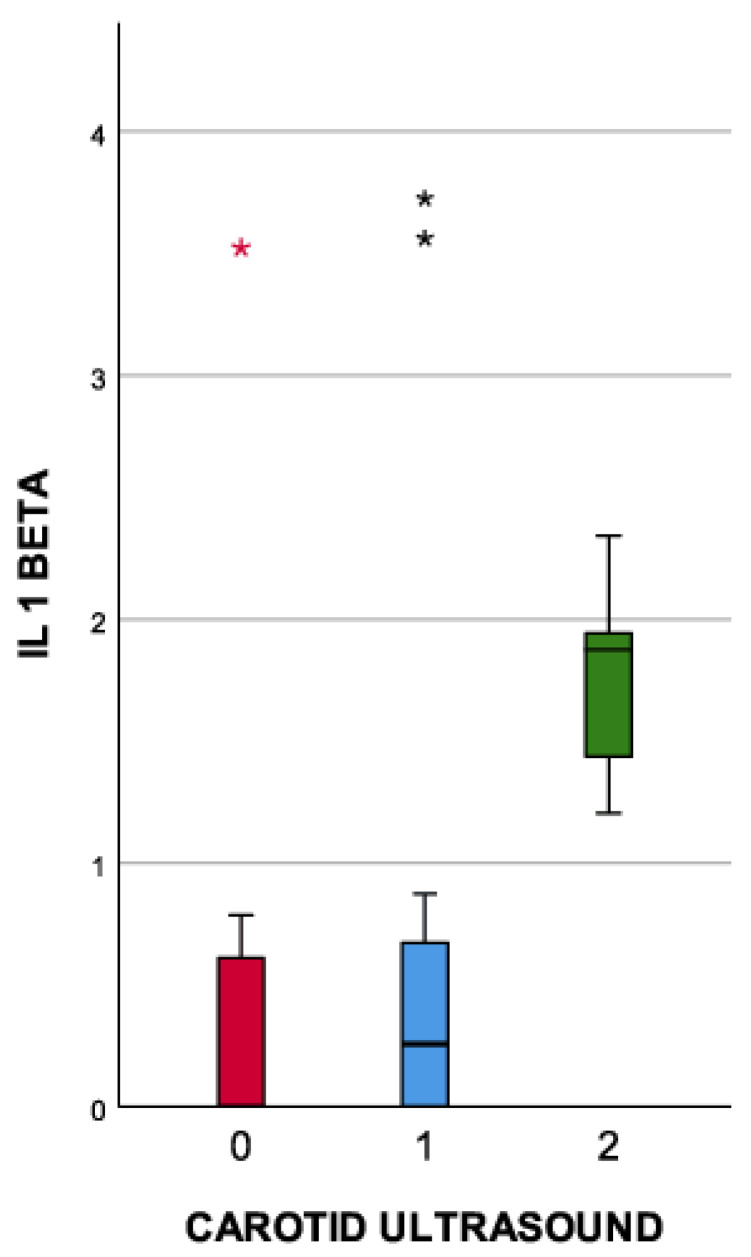
Correlation between the IL-1 beta plasma concentration (pg/mL) and the carotid ultrasound (extreme outliers are marked with an asterisk (*) on the boxplot).

Figure 3 illustrates that both measurements of the IL-1 beta value (day 1 and day 7, at the onset of the symptoms) were positively correlated with the degree of atherosclerotic lesions of the carotid artery, with Spearman correlation indexes of 0.529 (first measurement) and 0.653 (second measurement).

By considering the type of stroke, the patients enrolled in the study were assigned to three groups:

1. Lacunar stroke—patients with cerebral ischemic lesions smaller than 10 mm.
2. Anterior Vascular Territory (AVT)—patients with cerebral ischemia in the anterior vascular territory.
3. Posterior Vascular Territory (PVT)—patients with cerebral ischemia in the posterior vascular territory.

The initial value of the IL-1 beta showed elevated levels (>5 pg/mL) in all three types of ischemic stroke, with the smallest values being registered in the lacunar stroke group, followed by the PVT group. The AVT-type ischemic strokes were correlated with the highest levels of IL-1 beta.

Table 2 shows us how we can evaluate and follow up the patients with a moderate carotid stenosis. The cut-off value of 0.964 in the second measurement of the IL-1 beta plasma concentration defines the difference between severe and moderate carotid stenosis in the study group.

**Figure 4:**
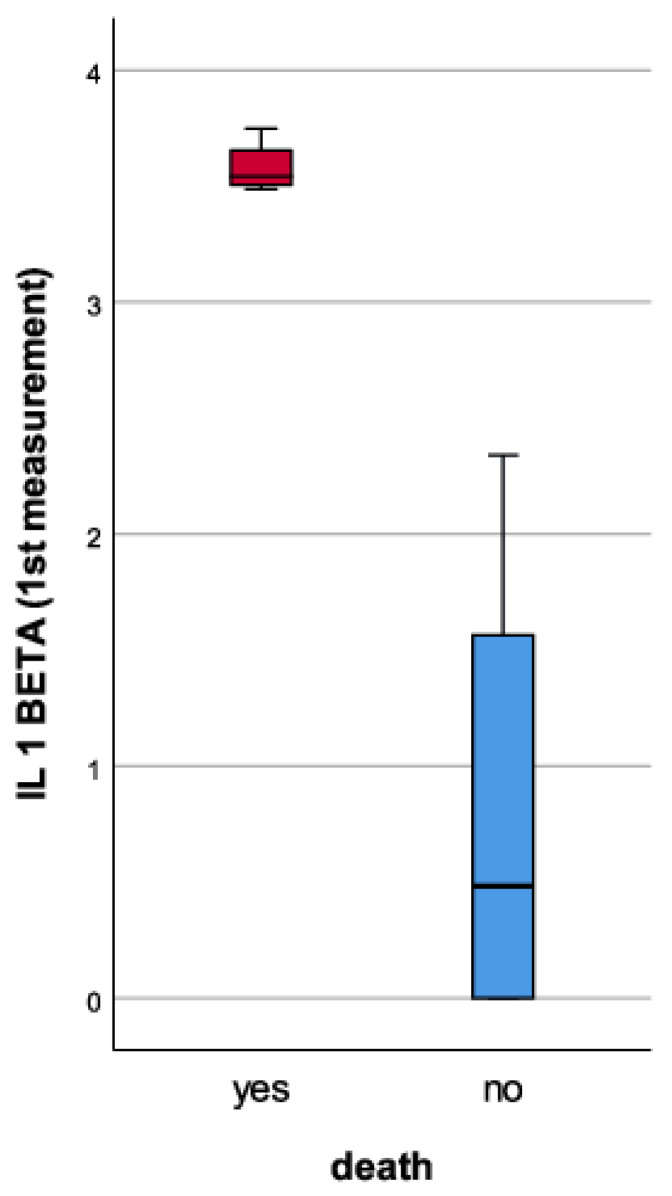
The IL-1 beta plasma concentration (pg/mL)’s correlation with patient mortality.

Furthermore, the blood levels of IL-1 beta were positively correlated with patient mortality (*p* value < 0.001). Figure 4 highlights how the first measurement of the IL-1 beta was correlated with patients’ death (6 out of the 56 patients enrolled in the study died—10.7%). At the same time, the first measurement of the IL-1 beta correlated positively and in a directly proportional way with the second measurement of the biomarker.

## 4. Discussion

IL-1 beta is a proinflammatory cytokine with a controversial role in the pathology of ischemic stroke according to the inconsistent results of previous clinical studies [11].

Recent studies have demonstrated that IL-1 beta stimulates the secretion of proinflammatory mediators, such as TNF-alpha cytokines and IL-6, as well as the secretion of adhesion molecules. At the same time, this cytokine is involved in astrocyte activation, which leads to the production of survival-promoting factors and, according to the literature, has a role in the development of risk factors for ischemic stroke, such as atherosclerotic lesions [12,13].

Clinical studies designed similarly to ours demonstrated that patients with ischemic stroke have elevated levels of IL-1 beta in the first 24 h from the beginning of the event (the same as the results from our study). Boutin et al. and Smith et al. concluded, after stage II of their single-center randomized studies, that administering IL-1 beta in mice led to the extension of cerebral ischemic lesions, and that mice with deficiency in IL-1 beta presented smaller volumes of cerebral infarction. At the same time, the authors of these studies highlighted that IL-1beta regulates the expression of IL-1 alpha; thus, IL-1beta is becoming a therapeutic target with multiple benefits. Anakinra is a recombinant human IL-1 receptor antagonist that binds to the IL-1 receptor, inhibiting the effect of both IL-1 beta and IL-1 alpha [13]. The IL-1 receptor antagonist, namely, IL-1Ra, has been tested in multiple clinical studies [14,15,16]. For instance, the patients who received the IL-1 receptors antagonist—Anakinra (100 mg loading dose, followed by 2 mg/kgc/h continuously via IV for 72 h)—had a reduced inflammatory response, with lower values of WBC count, hsCRP, and IL-1, probably due to a peripheral immunosuppression mechanism [17]. Likewise, the subcutaneous administration of IL-1Ra significantly reduced the inflammation level in patients included in the SCIL-STROKE study designed by Smith et al., without improving the clinical status at discharge (quantified with the RANKIN scale) [18,19].

The controversies concerning IL-1 beta gained momentum when clinical studies denied the role of this particular cytokine in the pathogenesis of ischemic stroke. At the same time, at least two studies reported IL-1 beta levels within the base levels at 12, 24, and 72 h after the onset of ischemic stroke [20]. Over the years, reports have been published suggesting that the genetic polymorphism of IL-1Ra is a risk factor for ischemic stroke and that inhibiting IL-1 beta can prevent or delay the onset of cerebral ischemia. Chiba et al. tried to inhibit IL-1 beta by giving mice a polyclonal antibody targeted against IL-1 beta (600 micrograms/day) [21]. The results were unexpected; the use of antibodies did slightly delay the onset of ischemic stroke in lab mice but without significance [21,22].

The discrepancies concerning the role of IL-1 beta at the onset of ischemic stroke arise from the design of the studies. Pre-clinical studies have shown good results, but clinical studies have demonstrated unfavorable, conflicting results. The differences are the result of the fact that the risk factors (smoking, alcohol consumption, stress, and age) cannot be reproduced in the laboratory, whereas the ischemic stroke in animals is induced by a simple, mechanical occlusion of the artery, with subsequent thrombus inflammation and micro thrombosis (with Treg cells activation). Other factors are the heterogeneity of ischemic strokes in human subjects and the fact that, in animal experimental models, complete arterial occlusion is followed by reperfusion, which is not the case in human subjects, except for patients with thrombectomy [13,23].

We have to underline the contribution of chronic inflammation, especially by IL-1 beta, to the pathologies considered risk factors for ischemic stroke, such as elevated blood pressure, obesity, infections, and atherosclerotic lesions.

Atherosclerotic lesions are recognized as part of a chronic inflammatory condition, defined by the formation of plaque in the arterial intima, which restricts blood flow and, at the same time, promotes the onset of ruptures and erosions at this level, favoring thrombotic occlusions [7,24]. IL-1 beta induces an inflammatory reaction in the endothelial cells, increasing the secretion of adhesion factors and chemokines. At the same time, this proinflammatory cytokine helps accumulate proinflammatory cells in the affected blood vessel, promoting their invasion in the intima and starting atheroma plaque formation [25].

One of the results of our study, namely, the correlation between the levels of IL-1beta with the severity of carotid atherosclerotic lesions, concurs with the literature’s data. In another similar study, Galea et al. compared the IL-1 beta levels with the coronarian atherosclerotic lesions in healthy patients and in patients with a cardiovascular pathology, concluding that there is a correlation between the IL-1 beta levels and the severity of atheromatosis [26,27]. At the same time, IL-1 beta levels are correlated with the production of more inflammatory mediators, such as cyclo oxygenase 2 (COX 2), leading to the formation of prostaglandins.

The synthesis of IL6 and matrix metalloproteinases (MMPs) are also mediated by IL-1 beta, leading to an elevation in the levels of acute phase reactants (C reactive protein, fibrinogen), with a role in the development of atherosclerotic lesions [28]. The metalloproteinases (MMP) 1, MMP8, and MMP13 are collagenases deeply related to the rupture of the fibrous cap of the atheroma plaque through their ability to break the collagen fibers. It is important to underline that IL-1 beta plays a significant role in the growth of already-settled atheroma plaque [29,30]. In about 80% of our patients, the plaques from the severely occluded arteries were old and fibrous, with a hyperechogenic appearance.

The pro-atherogenic feature of IL-1 beta has been demonstrated in many animal model studies. The chronic administration of IL-1 beta in pigs, in a study by Shimokawa et al. [31], resulted in elevated arterial intima-media thickness IMT, and, at the same time, the inhibition of IL-1 beta through the administration of IL-1Ra limited the development of atherosclerotic lesions [31]. Similarly, other studies, such as that by Chamberlain et al. [32], demonstrated that IL-1Ra deficiency can lead to neointima formation after endothelial lesions. The neointima formation was reduced through the administration of IL-1Ra or, in the case of IL-1 beta deficiency, without a causality between this and the IL-1 alpha levels [32].

Elhage et al. and Devlin et al. targeted the role of IL-1Ra, showing that its deficiency leads to transmural arterial inflammation, underlining the contribution of IL-1 in the initial stages of atherosclerotic lesion development [33,34].

The deficiency of IL-1 beta decreased the spontaneous development of atherosclerotic lesions in mice, while the transplantation of bone marrow with lower levels of IL-1 beta/IL-1 alpha resulted in a lower degree of diet-induced atherosclerotic lesions [35,36].

Recent studies have proven that obesity can interfere with the markers of inflammation, which is why it is necessary to highlight the fact that the majority of the patients included in our study who were diagnosed with moderate or severe atherosclerotic lesions were not obese (>75% had a BMI < 25). The research focused on Il-1 beta as the main biomarker and had different inclusion and exclusion criteria without considering the influence of atherosclerotic lesions (exclusion criteria were based on LDL and HDL cholesterol) [37]. At the same time, the studies classified patients into non-overweight, overweight, and obese groups according to their BMI, which can affect statistics given that BMI is considered a non-specific measure influenced by fat mass, lean tissue mass, and even height [38]. It is very difficult for comorbidities that can influence inflammation to be totally excluded from a study, so research in this field should be continued.

Studies conducted in recent years concluded that monoclonal antibodies targeting IL-1 beta are capable of reducing diet-induced atherosclerotic lesions in Apoe+ mice, hence the need for bigger cohort studies on human subjects in order to establish the molecule best suited (monoclonal antibodies and anti-inflammatory agents) to stop the development of atheromatous lesions. We review some of these studies (Cantos, Colcot, Cirt, Lodoco, and Convince) in Table 3 [39,40,41,42,43,44,45,46].

## 5. Limitations of the Study

It is necessary to emphasize that the study conducted by the authors of this article has certain limitations.

- The number of patients included in the study was relatively small. IL-1 beta should be included in studies with bigger cohorts before being considered a therapeutic target. However, the cohort was sufficient for it to be considered as a prognostic marker for ischemic stroke and atherosclerotic lesions of the carotid arteries.
- Measurements in long-term dynamics should be performed to determine what happens with inflammation, neurological status, and atherosclerotic lesions of the carotid arteries over time.
- Whether or not obesity and chronic medication can influence the IL-1 beta plasma concentration should be examined. Research on this subject should be developed and continued.

## 6. Conclusions

All these clinical studies have had promising results in stopping the evolution of cerebral ischemic lesions and atherosclerotic lesions of the carotid arteries, but the incidence of lethal infection was higher in the group treated with monoclonal antibodies or immunosuppressant medication. For this reason, this research should be continued and extended to other molecules targeting proinflammatory cytokines, such as IL-1 beta.

## Figures and Tables

**Table 1:** Description of the data included in the study (median—quartile 1–quartile 3).

AGE	74 (70–80)
GENRE	
F	28 (50%)
M	28 (50%)
NIHSS (ADMISSION)	7 (4–11)
NIHSS (DISCHARGE)	5 (2–10)
IL-1 BETA (24 h)	0.51 (0–1.56)
IL-1 BETA (DAY 7)	0 (0–1.59)
CHOLESTEROL	196 (55.79)
CAROTID ULTRASOUND	
0	14 (25%)
1	20 (35.71%)
2	22 (39.29%)

**Table 2:** The cut-off value for moderate and severe carotid stenosis.

	Cut-Off	Se	Sp	AUC	95% CI		SE	*p*-Value
IL-1 BETA (2nd measurement)	0.964	0.941	1	0.952	0.861	1	0.046	<0.001
IL-1 BETA (1st measurement)	1.0375	0.941	1	0.964	0.894	1	0.035	<0.001

**Table 3:** Studies conducted that imply future treatment for atherosclerotic lesions.

Trial Name	Study Design	Patient Number	Molecule	Results
The Canakinumab Anti-inflammatory Thrombosis Outcome Study CANTOS	Phase 3, multicenter, randomized, double-blind, placebo-controlled	10,061	Canakinumab—subcutaneous injection (50 mg, 150 mg, 300 mg) every 3 months vs. placebo	Benefits observed in the 150 mg treatment group
Colchicine Cardiovascular Outcomes Trial COLCOT	Phase 3 randomized, placebo-controlled	4745	Colchicine 0.5 mg/day vs. placebo	Benefits with serious adverse effects due to colchicine
Cardiovascular Inflammation Reduction Trial CIRT	Phase 3 multicenter, randomized, double-blind, placebo-controlled	4786	Oral methotrexate—1520 mg/weekly vs. placebo	No benefits
Low-Dose Colchicine Trial for Secondary Prevention of Cardiovascular Disease LODOCO	Phase 3 multicenter, randomized, double-blind, placebo-controlled	532	Colchicine 0.5 mg/day vs. placebo	Benefits, but major adverse effects
Low-Dose Colchicine Trial for Secondary Prevention of Cardiovascular Disease LODOCO2	Phase 3 multicenter, randomized, double-blind, placebo-controlled	5500	Colchicine 0.5 mg/day vs. placebo	Benefits observed during long-term follow-up
Colchicine for prevention of vascular inflammation in non-cardioembolic stroke CONVINCE	Phase 3 multicenter, open-label, placebo-controlled	2623	Colchicine 0.5 mg/day vs. placebo	Ongoing
Subcutaneous Interleukin-1 Receptor Antagonist in Ischemic Stroke SCIL-STROKE	Phase 2, single-center, double-blind, randomized, placebo-controlled	80	Anakinra	Benefits—reduced inflammation
Rilonacept to improve artery function in patients with atherosclerosis	Phase 2, single-center, double-blind, randomized, placebo-controlled	10	Rilonacept	Benefits—reduced inflammation

## Data Availability

The data presented in this study are available on request from the corresponding author. The data are not publicly available due to privacy and ethical concerns.
